# Identification of HLA-DRB1 association to adalimumab immunogenicity

**DOI:** 10.1371/journal.pone.0195325

**Published:** 2018-04-03

**Authors:** Mohan Liu, Jacob Degner, Justin Wade Davis, Kenneth B. Idler, Ahmed Nader, Nael M. Mostafa, Jeffrey F. Waring

**Affiliations:** 1 Pharmacogenetics and Human Genetics, Genomics Research Center, AbbVie Inc., North Chicago, IL, United States of America; 2 Clinical Pharmacology and Pharmacometrics, AbbVie Inc., North Chicago, IL, United States of America; Istituto di Ricovero e Cura a Carattere Scientifico Centro di Riferimento Oncologico della Basilicata, ITALY

## Abstract

Anti-drug antibody formation occurs with most biological agents across disease states, but the mechanism by which they are formed is unknown. The formation of anti-drug antibodies to adalimumab (AAA) may decrease its therapeutic effects in some patients. HLA alleles have been reported to be associated with autoantibody formation against interferons and other TNF inhibitors, but not adalimumab. We analyzed samples from 634 subjects with either rheumatoid arthritis (RA) or hidradenitis suppurativa (HS): 37 subjects (17 RA and 20 HS) developed AAA (AAA+) during adalimumab treatment and 597 subjects (348 RA, 249 HS) did not develop AAA (AAA-) during the clinical trials. Using next-generation sequencing-based HLA typing, we identified three protective HLA alleles (HLA-DQB1*05, HLA-DRB1*01,and HLA-DRB1*07) that were less prevalent in AAA+ than AAA–subjects (ORs: 0.4, 0.25 and 0.28, respectively; and P values: 0.012, 0.012 and 0.018, respectively) and two risk HLA alleles (HLA-DRB1*03 and HLA-DRB1*011) that were more abundant in AAA+ than AAA–subjects (ORs: 2.52, and 2.64, respectively; and P values: 0.006 and 0.019). Similar to the finding of Billiet *et al*. who found that carriage of the HLA-DRB1*03 allele was more prevalent in those with anti-infliximab antibodies (OR = 3.6, p = 0.002, 95% CI: [1.5,8.6]).), we found HLA-DRB1*03 allele was also more prevalent in anti-adalimumab positive (OR = 2.52, p = 0.006, 95% CI: [1.37,4.63]). The results suggest that specific HLA alleles may play a key role in developing AAAs in RA and HS patients treated with adalimumab.

## Introduction

Rheumatoid arthritis (RA) and hidradenitis suppurativa (HS) are autoimmune disorders that are mediated in part by overexpression of tumor necrosis factor-alpha (TNF-α)[1,2]. Adalimumab is a recombinant human IgG1 monoclonal antibody that binds specifically to TNF- α and neutralizes its biologic function by blocking its interaction with cell surface TNF- α receptors. Adalimumab has been used for several years for the treatment of RA, among other indications, and was recently approved for the treatment of HS[3]. Although adalimumab is a fully humanized monoclonal antibody, like other protein-based therapies, it exhibits immunogenicity in some patients [4,5]. Patients who develop anti-adalimumab antibodies (AAAs) may show reduced therapeutic response[6–9].

Clinically, different strategies have been developed to mitigate AAA formation. These include concomitant use of methotrexate and dosing at a higher frequency (i.e., weekly instead of every other week), both of which have been shown to reduce the rate of AAA formation[4,6]. However, the role of patient-related factors in determining AAA formation has not been determined. Formation of anti-drug antibodies against protein-based therapies has been shown to be associated with specific human leukocyte antigen (HLA) alleles[10]. The HLA-DRB1 alleles are associated with antibody formation against interferon-β and infliximab[4,6,11]. HLA alleles that may be associated with AAA formation in patients treated with adalimumab have not been reported. To thoroughly understand the relative immunogenicity of adalimumab in humans, we performed genotyping initially in HLA-class I and II regions in RA and HS subjects, and then followed by focusing on HLA class II regions in all subjects receiving subcutaneous adalimumab, to evaluate the association between specific HLA loci and AAA formation with adalimumab. Next-generation sequencing based HLA typing was performed in 634 subjects with either Rheumatoid Arthritis (RA) RA or Hidradenitis Suppurativa (HS): 37 subjects developed AAA (AAA+) during adalimumab maintenance treatment while 597 subjects never developed AAA (AAA-). We identified three protective alleles (HLA-DQB1*05, HLA-DRB1*01, and HLA-DRB1*07) that were less prevalent in AAA+ than AAA–subjects and two risk alleles (HLA-DRB1*03 and HLA-DRB1*011) that were more abundant in AAA+ than AAA–subjects. The results suggest that specific HLA alleles potentially play a key role in developing AAAs in RA and HS patients treated with adalimumab.

## Materials and methods

### Studies and subjects

Subjects were drawn from participants in four different phase III clinical trials. Samples from rheumatoid arthritis subjects were obtained from CONCERTO and MUSICA (ClinicalTrials.gov numbers NCT01185301 and NCT01185288, respectively) while samples from HS subjects were obtained as part of PIONEER I and PIONEER II (NCT01468207 and NCT01468233, respectively.). The authors had no access to any identifying participant information for this study. The details of these studies are summarized in Table 1 and have been reported previously[3,12,13]. The studies enrolled RA or HS subjects predominantly at sites in the US and Europe. RA subjects received subcutaneous injections of 40 mg adalimumab every other week for up to 24 weeks as well as weekly oral doses of methotrexate (2.5 to 20 mg), while HS subjects received adalimumab 40 mg subcutaneous injections every week or every other week for up to 36 weeks. The studies were conducted in accordance with Good Clinical Practice guidelines and ethical principles that have their origin in the Declaration of Helsinki. The protocols, protocol amendments, and informed consent forms were approved by the ethics committees and institutional review boards and written informed consent was obtained from each subject before any study-related procedures were performed.

**Table 1:** Overview of adalimumab RA and HS studies for HLA typing.

Study	Patients	Treatments	Number of AAA+ Samples Included in HLA Genotyping Analyses
CONCERTO NCT01185301	Early RA (diagnosis < 1year prior); biologic and MTX treatment-naïve (N = 395)	Oral MTX 2.5, 5, 10, or 20 mg weekly plus SC adalimumab 40 mg eow for 24 weeks	14 AAA+
MUSICA NCT01185288	RA with DAS28 [CRP] ≥ 3.2 at baseline; stable dose of MTX ≥ 15 mg/week for ≥ 12 weeks (N = 309)	Oral MTX 7.5 mg or 20 mg per week plus SC adalimumab 40 mg eow for 24 weeks	3 AAA+
PIONEER I NCT01468207	HS for ≥ 1 year; biologic treatment-naïve (N = 307)	SC adalimumab 40 mg ew or matching placebo for 12 weeks, followed by adalimumab 40 mg ew or eow or matching placebo for 24 weeks; all patients who received placebo in the first 12 weeks received adalimumab 40 mg ew thereafter	12 AAA+
PIONEER II NCT01468233	HS for ≥ 1 year;biologic treatment-naïve (N = 326)	SC adalimumab 40 mg ew or matching placebo for 12 weeks, followed by adalimumab 40 mg ew or eow or placebo for 24 weeks; all patients who received placebo in the first 12 weeks continued to receive placebo	8 AAA+

### Sample collection and analysis of adalimumab and AAA concentrations

Blood samples for determination of serum adalimumab and AAA concentrations were obtained by venipuncture in all studies. In CONCERTO and MUSICA, samples were obtained prior to dosing at Weeks 0 (Baseline), 2 (CONCERTO only), 4, 8, 12, 16, 20, and 24/26 or early termination. In PIONEER I and II, samples were obtained prior to dosing at Weeks 0, 4, 12, 16, 24, and 36. Serum adalimumab and AAA concentrations were measured using validated enzyme-linked immunosorbent assays (ELISA).

For AAA concentrations measured in samples from CONCERTO and MUSICA, the lower limit of quantitation (LLOQ) was 10.31 ng/mL in undiluted serum and 1.031 ng/mL in 10% diluted serum. The assay range was 1.031 to 25.0 ng/mL in diluted serum, the coefficient of variation (%CV) values were ≤ 8.8% and ≤ 9.4%, respectively, and the intra-run percent bias ranged from –1.0% to 1.1% and –5.0% to –2.2%, respectively, of their theoretical values. For AAA concentrations measured in samples from PIONEER I and II, the LLOQ was 10.00 ng/mL in undiluted serum. The assay range was 1.00 to 32.0 ng/mL in diluted serum, the CV values were ≤ 10.8% or 12.4%, respectively, and the intra-run percent bias values ranged from -14.4% to -0.3% and -14.7% to -2.6%, respectively, of their theoretical values.

Serum samples were analyzed for AAA using a validated ELISA method based on a double-antigen technique which detects antibodies against epitopes on the entire adalimumab molecule. Only samples with adalimumab concentration < 2 μg/mL were selected for AAA assay analysis. The LOQ for AAA in both studies was 0.5 ng/mL in diluted serum and 5 ng/mL in undiluted serum and the calibration range was 0.5–5.0 ng/mL. In-study QC samples supplemented with rabbit anti-idiotypic AAA (0.75–4.75 ng/mL) were included in the assay. The CV values were ≤ 5.45% and ≤ 5.31% for REVEAL and M02-528, respectively. Serum samples were considered AAA+ if measured AAA concentration was greater than 20 ng/mL, the signal was not reduced by 50% or more by addition of 10% human serum, and the serum sample was collected within 30 days after an adalimumab dose.

### HLA typing

To enrich for the strongest immunogenicity phenotype, AAA+ subjects with at least 2 samples that had AAA concentrations ≥ 100 ng/mL along with undetectable adalimumab concentrations were selected as AAA+ cases for HLA typing. Subjects with samples that did not meet the criteria for AAA+ were used as negative controls (AAA–). For the initial analyses of samples from subjects with RA, broad HLA genotyping (Class I and II) was performed using the Sanger method according to manufacturer's instructions (SeCore^®^ HLA typing kit, ThermoFisher Scientific, Grand Island, NY). In the follow-on analyses of all samples, HLA genotyping focused on the Class II DQB1 and DRB1 alleles was performed using next-generation sequencing (NGS) technology on a MiSeq System according to the manufacturer's instructions (Illumina, Inc., San Diego, CA). NGS were undertaken at AbbVie and Histogenetics (Ossining, NY, USA) using high-resolution HLA sequence-based typing (SBT)[14].

### Statistical analyses

For comparing HLA allele frequencies in AAA+ and AAA- subjects, two approaches were performed. In both approaches, we first label each allele as being derived from an AAA+ or an AAA- individual and test for deviations from the normal ratio of AAA+ to AAA-. The first approach treats each HLA allele type independently and tests the null hypothesis that AAA+: AAA- ratios are equal for the allele in question compared to the collection of all other alleles. Pseudo-counts of 0.5 were added to the final contingency table and Fisher’s Exact Tests were used to obtain P-values [15]. While the hypothesis tested in the first approach is of interest, each allele is compared to a different collection of other alleles so directly comparing odds ratios between alleles and adjusting for additional covariates is not possible. Thus, in a second approach, we fit a joint logistic regression model with the most common allele set as the reference (intercept) and estimated regression coefficients for all other alleles, which allows for a direct comparison between any two alleles if so desired. We tested models with HLA genotypes alone as predictor variables and compared the results of these models with models including sex, age, ethnicity, and disease type as additional covariates. Firth’s bias reduction was employed and significance was assessed with penalized profile likelihood and chi-squared test statistics as implemented in the logistf package in R[16].

## Results

In total, 365 RA subjects were included in the HLA typing analysis: 17 AAA+ cases (12 from CONCERTO and 3 from MUSICA) and 348 negative controls. Average AAA concentrations in these subjects ranged from 0.047 to 98.5 μg/mL. A total of 269 HS subjects were included in the HLA typing analyses: 20 AAA+ cases (12 from PIONEER-I and 8 from PIONEER-II) and 249 negative controls. Average AAA concentrations in these subjects ranged from 0.070 to 16.8 μg/mL.

### HLA class II allele analysis in RA and HS

**Fig 1:**
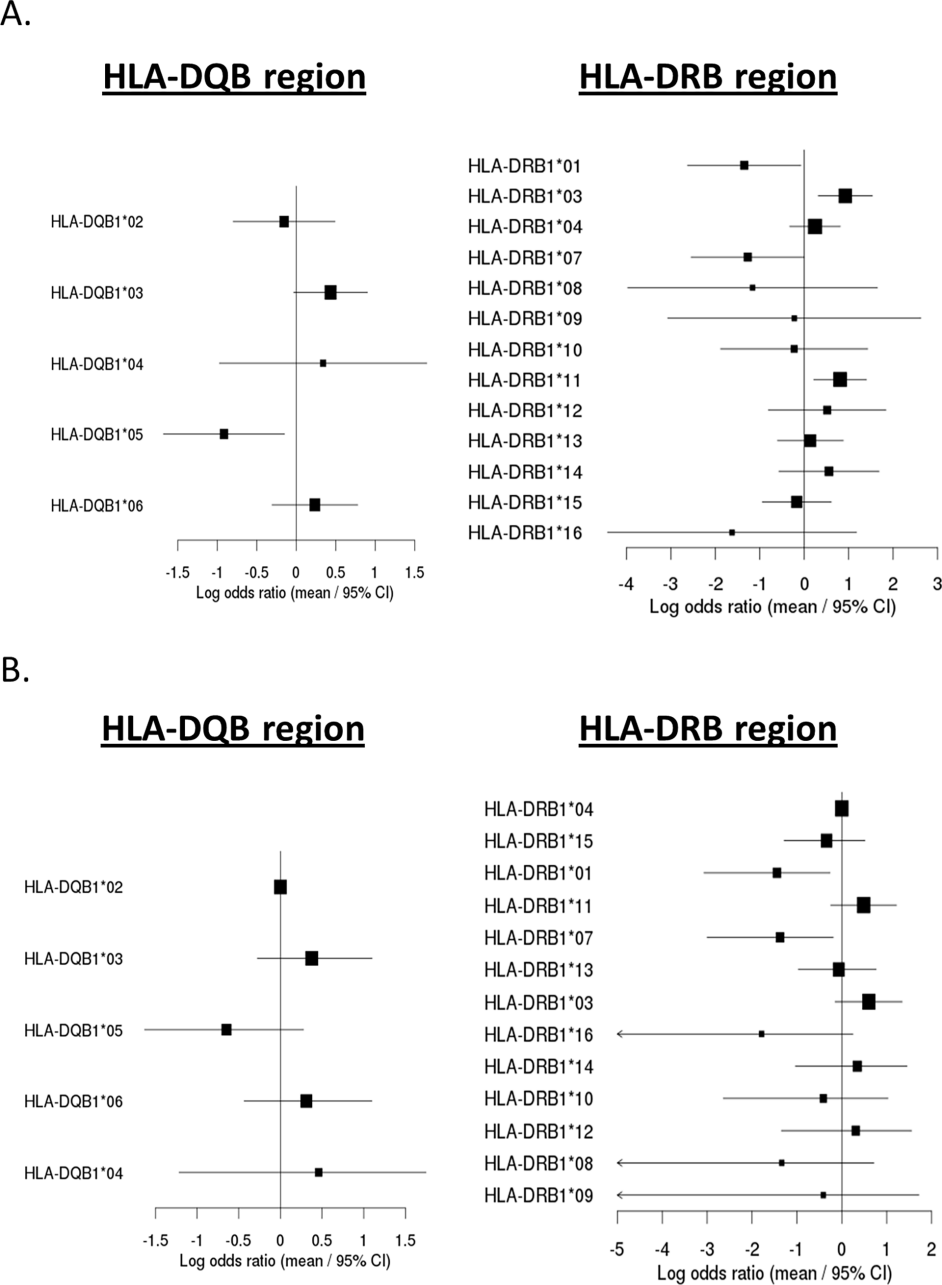
Forest plot of AAA formation (auto-antibody to adalimumab) according to the different HLA-DQB and DRB alleles in HS and RA by different tests. **(A)** HLA Class II DQB and DRB alleles effect of combined subjects in HS and RA by odds ratio and 95% confidence intervals (CIs) on AAA formation by Fisher’s Exact Test. **(B)** HLA Class II DQB and DRB alleles effect of combined subjects in HS and RA by odds ratio and 95% confidence intervals (CIs) on AAA formation by Fisher’s Exact Test.

Initial Sanger based HLA typing in both HLA-Class I and Class II regions was performed in a small subset of RA subjects (17 AAA+ and 20 AAA-) (data not shown). Based on the results, we focused on the HLA-Class II in the DQB1 and DRB1 regions for the full set of samples. NGS-based HLA typing analysis was performed using 37 AAA+ subjects (17 from RA and 20 from HS) plus 597 AAA- subjects (348 from RA and 249 from HS). When the results from both RA and HS subjects were combined with all AAA- controls available in these RA and HS trials, two statistical methods were utilized to analyze risk and protective alleles. In the first approach (Fisher’s exact) we compared each HLA allele to the collection of all other HLA alleles, and carriers of HLA-DRB1*01 and *07 were shown to be less prevalent in AAA+ than AAA- subjects (OR = 0.259, P = 0.012, 95% CI: [0.073–0.927] and OR = 0.281, P = 0.018, 95% CI: [0.078, 1.004], respectively). Carriers of HLA-DRB1*03 and *11 alleles were shown to be more prevalent in AAA+ than AAA- subjects (OR = 2.519, P = 0.006, 95% CI: [1.37, 4.63] and OR = 2.24, P = 0.019, 95% CI: [1.240, 4.045], respectively) (Fig 1A and Table 2). However, in the HLA-DQB region, HLA-DQB1*05 was shown to be less prevalent in AAA+ than AAA- subjects (OR = 0.400, P = 0.012, 95% CI: [0.186–0.862].

**Table 2:** HLA typing results for AAA formation in extension study in HLA-DQB1 and HLA-DRB1 regions for both RA and HS subjects by Fisher’s exact test.

HLA Allele	95% CI		OR	P value
	Lower	Upper		
HLA-DQB1*02	0.450	1.636	0.86	0.638
HLA-DQB1*03	0.968	2.467	1.55	0.084
HLA-DQB1*04	0.378	5.232	1.41	0.694
HLA-DQB1*05	0.186	0.862	0.40	0.012^§^
HLA-DQB1*06	0.735	2.181	1.27	0.460
HLA-DRB1*01	0.073	0.927	0.26	0.012^§^
HLA-DRB1*03	1.370	4.632	2.52	0.006^§^
HLA-DRB1*04	0.721	2.250	1.27	0.438
HLA-DRB1*07	0.078	1.004	0.28	0.018^§^
HLA-DRB1*08	0.019	5.185	0.31	0.394
HLA-DRB1*09	0.046	13.79	0.80	1.000
HLA-DRB1*10	0.152	4.175	0.80	1.000
HLA-DRB1*11	1.240	4.045	2.24	0.019^§^
HLA-DRB1*12	0.446	6.276	1.67	0.658
HLA-DRB1*13	0.548	2.407	1.15	0.841
HLA-DRB1*14	0.565	5.381	1.74	0.455
HLA-DRB1*15	0.389	1.837	0.85	0.707
HLA-DRB1*16	0.012	3.238	0.20	0.164

^§^ P value significance level is defined at 0.05; CI: confidence interval; OR: odds ratio

*: Allele

In the second approach, we used logistic regression with Firth’s bias reduction to jointly estimate odds ratios and significance when comparing each HLA allele to a common reference allele (with loss of generality, we used the most common allele as reference). In this analysis, carriers of HLA-DRB1*01 and *07 were shown to be less prevalent in AAA+ than AAA- subjects (OR = 0.236, P = 0.014, 95% CI: [0.046, 0.770] and OR = 0.253, P = 0.021, 95% CI: [0.050, 0.826]). Carriers of HLA-DRB1*03 and *11 alleles were consistent in direction with the previous approach but not significant (OR = 1.825, P = 0.115, 95% CI: [0.860, 3.836] and OR = 1.627, P = 0.191, 95% CI: [0.781, 3.375]) (Fig 1B and Table 3)., In the HLA-DQB region, HLA-DQB1*05 was less prevalent in AAA+ than AAA- subjects but this trend was not significant (OR = 0.55, P = 0.21, 95% CI: [0.21, 1.40]) (Fig 1B and Table 3). Framing the statistical model as a logistic regression also allowed us to assess the robustness of our findings to a range of potential covariates. We found that for all tested covariates (sex, age, ethnicity, and disease type), the estimates of effect size for all HLA alleles remained within 17% of their value without covariates (the maximum change occurred for HLA-DRB1*09 using disease type as a covariate). We found that the average change across all alleles and covariate choices was only 2%; that no direction of effect estimates changes; and that no classifications of which alleles were significant changed.

**Table 3:** HLA typing results for AAA formation in HLA-DQB1 and HLA-DRB1 regions in extension study for both RA and HS subjects by logistic model test.

HLA Allele	95% CI		OR	P value
	Lower	Upper		
HLA-DQB1*02	NA	NA	1.00	NA
HLA-DQB1*03	0.76	3.02	1.46	0.26
HLA-DQB1*04	0.29	5.63	1.55	0.56
HLA-DQB1*05	0.21	1.40	0.55	0.21
HLA-DQB1*06	0.64	2.99	1.36	0.42
HLA-DRB1*01	0.046	0.770	0.24	0.014^§^
HLA-DRB1*03	0.860	3.836	1.83	0.115
HLA-DRB1*04	1.000	1.000	1.00	NA
HLA-DRB1*07	0.050	0.826	0.25	0.021^§^
HLA-DRB1*08	0.002	2.043	0.26	0.254
HLA-DRB1*09	0.005	5.568	0.66	0.766
HLA-DRB1*10	0.071	2.797	0.66	1.000
HLA-DRB1*11	0.781	3.375	1.63	0.191
HLA-DRB1*12	0.260	4.727	1.67	0.620
HLA-DRB1*13	0.379	2.148	0.94	0.876
HLA-DRB1*14	0.356	4.273	1.41	0.588
HLA-DRB1*15	0.277	1.676	0.71	0.446
HLA-DRB1*16	0.001	1.282	0.17	0.098

^§^ P value significance level is defined at 0.05; CI: confidence interval; OR: odds ratio; NA: not available

*: Allele

## Discussion

This study aimed to investigate if HLA alleles are associated with AAA formation. HLA typing analyses were performed to determine whether there is an association between genetic loci in the HLA region and formation of AAAs in subjects with RA or HS. The results of the present analyses suggest that in subjects with RA or HS, carriage of the HLA-DRB1*01 and HLA-DQB1*05 alleles may protect against AAA formation whereas carriage of the HLA-DQB1*03, HLA-DRB1*04, and HLA-DRB1*03 alleles may increase the risk of AAA formation. The results of our analyses for the association of the HLA-DRB1 allele with AAA formation are consistent with recent reports of the association of HLA-DRB1 alleles and anti-drug antibody formation to infliximab and interferon-β[4,17–19]. In patients with IBD treated with infliximab for at least 2 years, carriage of the HLA-DRB1*03 allele was more prevalent in patients who developed antibodies to infliximab than those who did not (OR: 3.6, 95% CI: 1.5 to 8.6) and HLA-DRB1*13 was less prevalent in patients who developed antibodies to infliximab than those who did not (OR: 0.44, 95% CI: 0.22 to 0.91)[4]. In this study for subjects with RA or HS receiving treatment with adalimumab, carriage of the HLA-DRB1*03 allele were shown to confer an increased risk for developing antibodies to Adalimumab (Table 4). Similarly, adalimumab present in the same arginine at position 74 and glutamate at position 71, which were also associated with anti-antibody formation in Infliximab[20] (S1 Fig). The hypothesis from this finding was the possible functional role for the HLA-DRB1*03 allele in antibody formation by showing that the presence of arginine at position 74 and the absence of glutamate at position 71 of the B1-chain encoded by HLA-DRB1 were associated with infliximab antibody formation. Both amino acid positions are located in the peptide binding groove of the HLA-DR complex [21].

**Table 4:** Comparison the Allelic association of HLA-DRB1*03 between published anti-infliximab antibody formation in IBD patients and in our AAA formation in RA and HS patients. The Allelic Association of HLA-DRB1*03 to Anti-infliximab Formation(ATI) among 76 ATI + Subjects and 116 ATI–Subjects [22], and in Formation in RA and HS among 37 AAA + Subjects and 597 AAA- Subjects by Fisher’s Exact Test and Logistic Model Test.

HLA-DRB1*03	Cases(76)	Controls(116)	OR	P value	95% CI
anti-infliximab	0.13 (20)	0.04 (9)	3.6	0.002^§^	1.5–8.6
Anti-adalimumab	Cases(37)	Controls(597)	OR	P value	95% CI
Fisher’s Exact	0.2 (14)	0.093 (110)	2.52	0.006^§^	1.37–4.63

^§^ P value significance level is defined at 0.05; CI: confidence interval; OR: odds ratio

*: Allele

The specific HLA-DRB1 and-DQB1 alleles identified in our analyses have been implicated in antibody formation in other settings. The HLA-DRB1*01 allele was protective against AAA formation in our analyses of subjects with RA or HS and has been shown to be protective against anti-cyclic citrullinated peptide antibodies in RA women treated with hormone replacement therapy[23]. In addition, the HLA-DRB1*04 allele, which conferred increased risk of AAA formation, has been reported as the most significant contributor to the risk of antibody formation in RA[11]. Finally, the HLA-DRB1*03 allele, which also conferred increased risk of AAA formation, has been shown to be associated with autoantibody formation in lupus[24]. Investigation of the clinical relevance of HLA typing for adalimumab dosing and AAA formation may be warranted in future studies.

## Conclusions

HLA typing analysis of RA and HS subjects who received an adalimumab treatment, revealed that specific HLA-DRB1 and HLA-DBQ1 alleles are associated with risk of or protection against AAA formation, respectively. HLA-DRB1*03 is independently predictive of AAA formation in RA and HS subjects with adalimumab treatment and in IBD subjects with infliximab treatment. These results provide additional insight into underlying reasons for variable immunogenicity response across different patients treated with anti-TNF therapy.

## Supporting information

S1 Fig Sequence comparison between infliximab and adalimumab. The CDRs are highlighted by black frames and labeled. The residues that platy crucial roles in the antibody-antigen interaction are framed with blue frames. Adopted from Hu S *et al*. Comparison of the inhibition mechanisms of adalimumab and infliximab in treating tumor necrosis factor associated diseases from a molecular view.
(TIF)
