# Supplementary figures and images for: Identification of HLA-DRB1 association to adalimumab immunogenicity
Source: PLoS One. 2018 Apr 3;13(4):e0195325. doi: 10.1371/journal.pone.0195325 (PMC5882140; doi:10.1371/journal.pone.0195325)

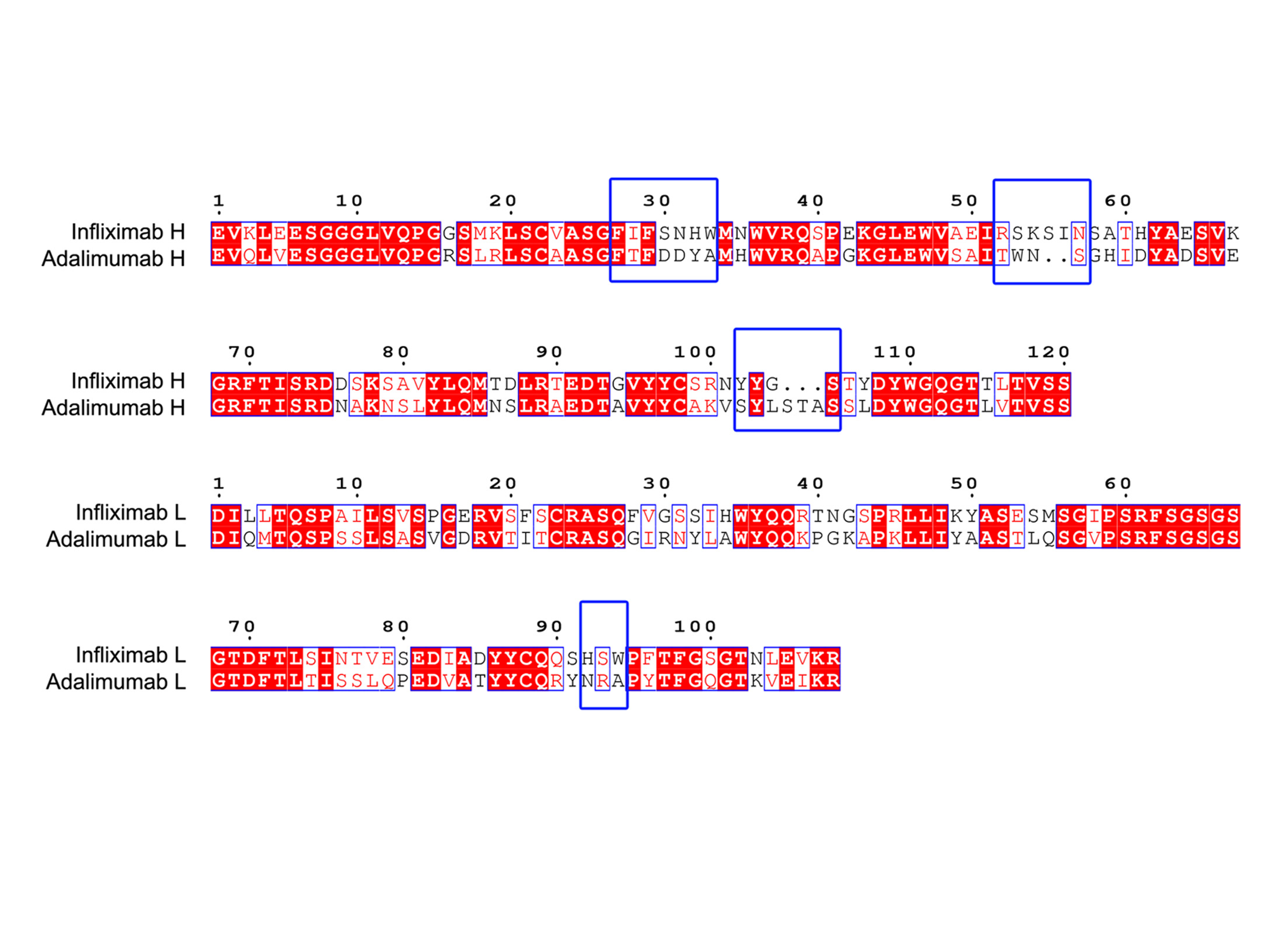

Supplement: S1 Fig — The CDRs are highlighted by black frames and labeled. The residues that platy crucial roles in the antibody-antigen interaction are framed with blue frames. Adopted from Hu S et al. Comparison of the inhibition mechanisms of adalimumab and infliximab in treating tumor necrosis factor associated diseases from a molecular view. (TIF) [file pone.0195325.s001.tif]
